# Incidence, management, and prognosis of post-ischaemic ventricular septal defect: Insights from a 12-year tertiary centre experience

**DOI:** 10.3389/fcvm.2022.1066308

**Published:** 2022-12-06

**Authors:** Henri Treille de Grandsaigne, Frédéric Bouisset, Jean Porterie, Caroline Biendel, Bertrand Marcheix, Olivier Lairez, François Labaste, Meyer Elbaz, Michel Galinier, Clément Delmas

**Affiliations:** ^1^Intensive Cardiac Care Unit, Rangueil University Hospital, Toulouse, France; ^2^Cardiology Department, Rangueil University Hospital, Toulouse, France; ^3^Cardiovascular Surgery Department, Rangueil University Hospital, Toulouse, France; ^4^Department of Anesthesiology, Intensive Care Medicine and Perioperative Medicine, Rangueil University Hospital, Toulouse, France

**Keywords:** acute cardiac care, acute coronary syndrome, mortality, complication, epidemiology

## Abstract

**Background:**

Among mechanical complications of acute myocardial infarction, ventricular septal defect (VSD) is uncommon but still serious. The evolution of emergency coronary revascularisation paradoxically decreased our knowledge of this disease, making it even rarer.

**Aim:**

To describe ischaemic VSD incidence, management, and associated in-hospital and 1-year outcomes over a 12-years period.

**Methods:**

A retrospective single-centre register of patients managed for ischaemic VSD between January 2009 and December 2020.

**Results:**

Ninety-seven patients were included representing 8 patients/ years and an incidence of 0.44% of ACS managed. The majority of the patients were 73-years-old males (*n* = 54, 56%) with STEMI presentation (*n* = 75, 79%) and already presented with Q necrosis on ECG (*n* = 70, 74%). Forty-nine (51%) patients underwent PCI, 60 (62%) inotrope/vasopressors infusion, and 70 (72%) acute mechanical circulatory support (IABP 62%, ECMO 13%, and Impella® 3%). VSD surgical repair was performed for 44 patients (45%) and 1 patient was transplanted. In-hospital mortality was 71%, and 86% at 1 year, without significant improvement over the decade. Surgery appears to be a protective factor [0.51 (0.28–0.94) *p* = 0.003], whereas age [1.06 (1.03–1.09), *p* < 0.001] and lactate [1.16 (1.09–1.23), *p* < 0.001] were linked to higher 1-year mortality. None of the patients that were managed medically survived 1 year.

**Conclusion:**

Post-ischaemic VSD is a rare but serious complication still associated with high mortality. Corrective surgery is associated with better survival, however, timing, patient selection, and a place for mechanical circulatory support need to be defined.

## Introduction

Ventricular septal defect (VSD) is an uncommon but serious mechanical complication of myocardial infarction (MI), most often associated with an acute ST-elevation MI (STEMI), related to the acute occlusion of a major epicardial vessel. If its prevalence was relatively high during the pre-thrombolytic era, 1 to 2% (1), technical improvements in coronary revascularisation and the development of pre-hospital networks have made it even rarer (0.2%) (2, 3). VSD is the least rare mechanical complication (0.21%) of MI, compared to mitral regurgitation due to papillary muscle ischaemic rupture (0.05%), and free wall rupture (0.01%) (3). In addition, whatever the mechanical complication, its occurrence is five times more likely in STEMI vs. non-STEMI (3). The admissible pathophysiological argument is that longer and deeper ischaemia would produce such necrosis that it has noteworthy anatomical repercussions (4).

Despite its rarity, ischaemic VSD is still associated with high mortality if medically managed (above 90% mortality at 30 days) and its management remains challenging (1, 5). The only curative therapy is then surgical septum repair, mostly by VSD patch closure using the double ventriculostomy technique, which remains a high-risk procedure mainly due to recent infarction, depressed ventricular function, and often precarious hemodynamics. VSD repair is still associated with high postoperative mortality (33% to 61 %) (1, 5–7). Moreover, surgery timing is also pivotal (8), as suggested by the higher mortality observed among the patients undergoing surgery before day 7 (54% vs. 17%) (9), even if these observations are probably subject to selection bias. Finally, discussion on the timing of surgery and the better results observed in the case of delayed surgery raises the question of the waiting conditions. During this time frame, physicians should be able to stabilize the patient's hemodynamics, correct any organ failure, and unload the ventricles in order to promote myocardial recovery before VSD correction. Acute mechanical circulatory support (aMCS) is thus often discussed for this purpose; however, the type and timing of its deployment are still a matter of debate (10).

We aimed to assess the evolution of incidence, management, and prognosis of ischaemic VSD during the last 10 years in a large tertiary center in western Europe.

## Methods

### Population

All the consecutive patients managed for ischaemic VSD at Toulouse University Hospital between 01/01/2009 and 31/12/2020 were retrospectively included in this study. The patients were found *via* the local PMSI (Programme de Médicalisation des Systèmes d′Information) (ICD codes 10 X and Y), with a retrospective control carried out using the intensive cardiac care unit register. According to the French ethical and regulatory law, retrospective studies based on the exploitation of usual care data do not need to be submitted to an ethical committee but have to be covered by the reference methodology of the French National Commission for Informatics and Liberties (CNIL). After evaluation and validation by the data protection officer and according to the General Data Protection Regulation, this study completed all the criteria and was registered in the register of retrospective studies of the Toulouse University Hospital (number RnIPH 2020–115) and covered by the MR-004 (CNIL number: 2206723 v 0).

### Data collection

Clinical data were collected at admission and during hospitalization, together with a previous history of heart disease, cardiovascular risk factors, and comorbidities. Transthoracic echocardiography (TTE) was performed in all cases and data obtained were collected (left and right ventricle diastolic and systolic function, size and localization of the VSD). Biological monitoring included blood gas and arterial lactate, prothrombin ratio, NT-pro-BNP, aspartate aminotransferase, alanine aminotransferase, total bilirubin and creatinine levels, and estimated glomerular filtration rate. The EuroSCORE II was calculated for all patients.

### Statistics

Continuous variables were expressed as means ± standard deviation or as medians with interquartile ranges (IQR) when not normally distributed. Nominal variables were expressed in numbers and percentages. The association between the mean values of continuous variables was assessed using the Mann-Whitney rank sum test or Student′s t-test when appropriate. Nominal variables were assessed by the χ^2^ test or Fisher′s exact test when appropriate. The patients were separated into two groups based on in-hospital (Supplementary Table S1) or 1-year (Table 1) mortality. Regression analysis was performed using variables with a *p*-value < 0.05 to analyse variables associated with the criteria of mortality with results reported as odds ratios (OR) with 95% confidence intervals (CI) for in-hospital mortality. A Cox analysis was performed to analyse the factors associated with 1-year mortality and the results were reported as a hazard ratio (HR) with 95% CI (Table 2). A *p*-value inferior to 0.05 was considered significant. Stata® (14.2 version) software was used for statistical analyses.

**Table 1:** Description of the population at admission, management, and outcomes according to their 1-year mortality.

	**Global population** **(*n* = 97)**	**Non-survivors at 1 year** **(*n* = 83)**	**Survivors at 1 year** **(*n* = 14)**	***p*-value**
Age, years old	73 +/- 11	74 +/- 9	63 +/- 13	<0.001
Male sex, *n* (%)	54 (55.7)	45 (54.2)	9 (64.3)	0.48
**Cardiovascular risk factors, *n* (%)**			
Smokers (*n* = 95)	37 (38.9)	27 (33.3)	10 (71.4)	< 0.01
Diabetes (*n* = 96)	30 (31.3)	26 (31.7)	4 (28.6)	0.82
Dyslipidemia (*n* = 95)	32 (33.7)	26 (32.1)	6 (42.9)	0.43
Hypertension (*n* = 95)	53 (55.8)	45 (55.6)	8 (57.1)	0.91
Obesity (BMI > 30) (*n* = 90)	17 (18.9)	12 (15.8)	5 (35.7)	0.08
**Medical history, *n* (%)**				
Ischaemic cardiomyopathy (*n* = 94)	9 (9.6)	8 (10)	1 (7.1)	0.73
PAD (*n* = 95)	7 (7.4)	6 (7.4)	1 (7.1)	0.97
CKD (*n* = 95)	6 (6.3)	4 (4.9)	2 (14.3)	0.18
**Clinical characteristics at admission, n (%)**				
Right HF signs (*n* = 87)	35 (40.2)	28 (37.8)	7 (53.9)	0.27
Left HF signs (*n* = 92)	54 (58.7)	47 (60.3)	7 (50)	0.47
Hemodynamics instability (*n* = 97)	53 (54.6)	47 (56.6)	6 (42.9)	0.33
**Electrocardiogram at admission**, ***n*** **(%)**				
STEMI (*n* = 95)	75 (79)	66 (81.5)	9 (64.3)	0.14
Q wave	70 (73.7)	60 (74.1)	10 (71.4)	0.83
**Angiocoronarography, *n* (%)**				
Culprit lesion location (*n* = 91)				0.75
LAD	51 (56)	43 (55.8)	8 (57.1)	
RCA	37 (40.7)	31 (40.3)	6 (42.9)	
Cx	3 (3.3)	3 (3.9)	0 (0)	
*Ad-hoc* revascularisation	49 (50.5)	42 (50.6)	7 (50)	0.96
Significant other coronary artery disease (*n* = 91)				0.63
One-vessel disease	37 (40.7)	30 (39)	7 (50)	
Two-vessel disease	33 (36.3)	28 (36.4)	5 (35.7)	
Tri-vessel disease	21 (23.1)	19 (24.7)	2 (14.3)	
**Biology at admission**				
Lactates (mmol/l) (*n* = 84)	2.65 [1.75–4.7]	2.8 [1.8–5.4]	1.4 [1.2–2.2]	< 0.01
PTT (%) (*n* = 91)	66 +/- 19	65 +/- 20	73 +/- 12	0.13
Hepatic cytolysis (n x Normale) (*n* = 90)	6 (2–17)	7 (2–18)	3 (2–11)	0.19
Total bilirubin (mmol/l) (*n* = 88)	21.8 +/- 13.6	22.8 +/- 14.4	16.4 +/- 5.2	0.10
pH (*n* = 91)	7.39 +/- 0.12	7.37 +/- 0.13	7.45 +/0.06	0.06
Troponin (n x Normale) (*n* = 95)	420 [217–1,192]	427 [227–1,192]	427 [227–1,192]	0.24
Natriuretic peptid (n x Normale) (*n* = 66)	20 (9–49)	23 (10–53)	9 (7–19)	0.03
eGFR (MDRD) ml/min/1.73 m^2^ (*n* = 96)	43 [28–73]	40 [28–69]	66 [37–85]	0.08
**Echocardiography (TTE or TOE), *n* (%)**				
LVEF (%) (*n* = 96)	44 +/- 13	44 +/- 13	40 +/- 15	0.28
RV dilatation (*n* = 88)	50 (56.8)	39 (52.7)	11 (78.6)	0.07
RV dysfunction (*n* = 92)	44 (47.8)	38 (48.7)	6 (42.9)	0.68
**VSD characteristics**				
Size by TTE (mm) (*n* = 67)	14 +/- 8	13 +/- 7	16 +/- 11	0.23
Surgical size (mm) (*n* = 38)	23 +/- 13	23 +/- 14	26 +/- 10	0.51
VSD localization (*n* = 96)				0.70
Basal	28 (29.2)	25 (30.5)	3 (21.4)	
Median	16 (16.7)	14 (17.1)	2 (14.3)	
Apical	52 (54.3)	43 (52.4)	9 (64.3)	
EuroSCORE 2 (*n* = 95)	42.4 +/- 20.7	44.9 +/- 19.7	28 +/- 20.9	< 0.01
**VSD management**				
Inotrops or vasopressors	60 (61.9)	56 (67.5)	4 (28.6)	< 0.01
IABP	60 (61.9)	48 (57.8)	12 (85.7)	0.04
VA-ECMO	13 (13.4)	12 (14.5)	1 (7.1)	0.45
Impella®	3 (3.1)	2 (2.4)	1 (7.1)	0.34
All acute MCS	70 (72.2)	58 (69.9)	12 (85.7)	0.22
Surgical closure	44 (45.4)	32 (38.6)	12 (85.7)	< 0.01
Percutaneous closure	4 (4.1)	4 (4.8)	0 (0)	0.40
Heart transplantation	1 (1)	0 (0)	1 (7.1)	0.01
**Length of stay (days) (*n* = 96)**				
ICU/ICCU LOS	7 (3–12)	6 (3–10)	16 (11–27)	< 0.01
Total LOS	8 (3–16)	6 (3–12)	23 (16–46)	< 0.01

BMI, body mass index; CKD, chronic kidney disease; Cx, circonflex coronary artery; eGFR, estimated glomerular filtration rate; HF, heart failure; IABP, intra-aortic balloon pump; ICU, intensive care unit; ICCU, intensive cardiac care unit; LAD, left anterior descending artery; LOS, length of stay; LVEF, left ventricular ejection fraction; MCS, mechanical circulatory support; PAD, peripheral arterial disease; PTT, prothrombin time; RCA, right coronary artery; RV, right ventricle; TOE, transesophagal echocardiography; TTE, transthoracic echocardiography; VA-ECMO, veno-arterial extracorporeal membrane oxygenation; VSD, ventricular septal defect.

**Table 2:** Factors associated with in-hospital mortality by Cox regression analysis.

	**Univariate analysis**			**Multivariate analysis**		
	**OR**	**CI 95%**	***p* value**	**OR**	**CI 95%**	***p* value**
Age (for each supplementary year)	1.06	[1.02–1.11]	< 0.01	1.06	[1.01–1.12]	0.02
Active smocker	0.42	[0.17–1.04]	0.06	-	-	-
Chronic renal failure	0.39	[0.07–2.06]	0.26	-	-	-
**Biology**						
Lactates (for each supplementary mmol)	1.35	[1.01–1.81]	0.04	-	-	-
TP (for each supplementary %)	0.97	[0.95–1.01]	0.10	-	-	-
Hepatic cytolysis > 20 N	3.40	[1.06–10.9]	0.04	-	-	-
**Management**						
VSD surgical repair	0.09	[0.03–0.28]	< 0.01	0.14	[0.04–0.45]	< 0.01
Amine use	3.78	[1.50–9.49]	< 0.01	3.08	[1.22–11.4]	< 0.01

CI, confidence interval; HR, hazard ratio; N, normal; PT, prothrombin time; VSD, ventricular septal defect.

## Results

### Population

**Figure 1:**
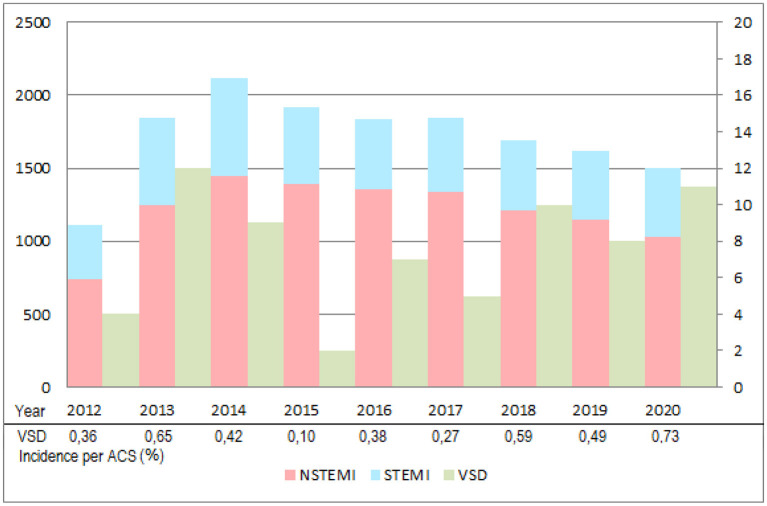
Annual numerical comparison of acute coronary syndromes (Non-STEMI and STEMI) and ischemic VSD managed between June 2012 and December 2020. Number of Non-STEMI (red) and STEMI (blue) per year are reported on the left axis although VSD (green) is reported on the right. Patients before January 2012 were nor reported since an old data system did not permit a systematic counting of all consecutive cases. ACS, acute coronary syndrome; NSTEMI, non-ST-elevated myocardial infarction; STEMI, ST-elevated myocardial infarction; VSD, ventricular septal defect.

Over a period of 12 years, a total of 97 patients with ischaemic VSD were included (Table 1), with a mean of 8 per year. Figure 1 shows VSD cases by year in comparison with STEMI and non-STEMI in our center and associated VSD incidence which varied between 0.10 % (2015) to 0.73 % of ACS (2020).

Patients were predominantly male (*n* = 54, 56%) with a mean age of 73 ± 11 years old. Cardiovascular risk factors were frequent but ischaemic cardiomyopathy was previously known for only 9 patients (9.6%). At admission 44 patients (45%) were in cardiogenic shock, 57 (59%) presented with left heart failure, and 39 (40%) with right heart failure signs. An ECG showed a Q wave of sequelae for 70 (74%). TTE showed a left (LVEF 44%) and right ventricles dysfunction (RV dilation 57% and/or dysfunction 48%). The VSD was apical for 52 patients (54%), with a mean size of 14 mm. Patients had one-vessel disease in 40% of the cases and the left anterior descending artery was the culprit vessel for 50 patients (56%).

### Management

An *ad hoc* revascularisation was performed for 49 patients (51%), despite a mean delay from initial symptoms of 52 h. Inotrope/vasopressor support was needed for 60 patients (62%) and mechanical circulatory support for 70 (72%), mainly by IABP (*n* = 68, 86%). The patients supported by an aMCS were younger (71 +/- 10 vs. 77 +/- 9 yo, *p* = 0.01), with less peripheral artery disease (2.9 vs. 20%, *p* = 0.002), with larger VSD (26 +/- 13 vs. 15 +/- 5 mm, *p* = 0.03). They were also more managed by inotropes/vasopressors (71.4 vs. 37.4%, *p* = 0.002) with more surgical closures attempted (51.4 vs. 29.6%, *p* = 0.05) and a higher length of stay in the critical care unit (9 vs. 3 days, *p* = 0.002) (Supplementary Table S1). For 44 patients (45%), a surgical VSD repair was performed and for 1 patient (1%) a heart transplant was required, with a mean delay to the procedure of 10.6 days. A percutaneous closure technique was attempted for 4 patients (4%) considered to be at prohibitive risk for surgery.

### Outcomes

**Figure 2:**
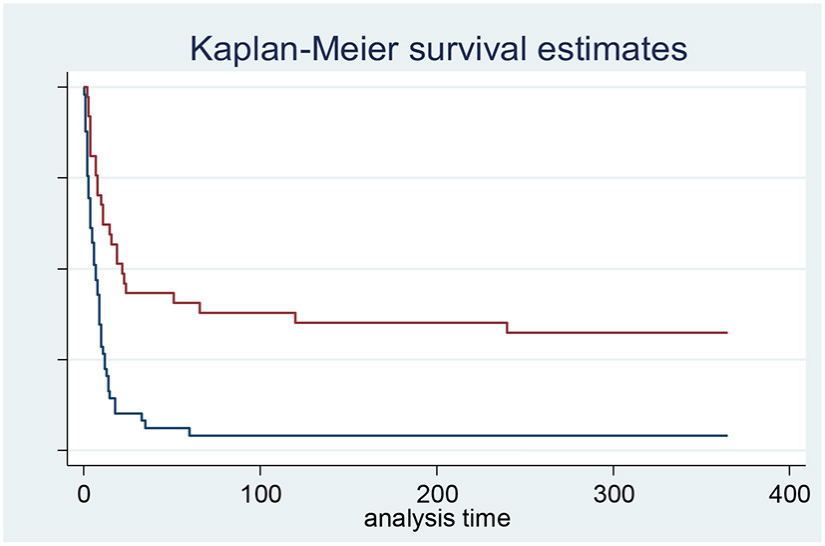
Ischemic ventricular septal defect survival according to the realization of surgical repair (red curve) or not with only medical management (blue curve). Log-rank test *p*-value < 0.001. On 97 patients. 11 patients were lost of follow-up and not included in survival analysis.

**Figure 3:**
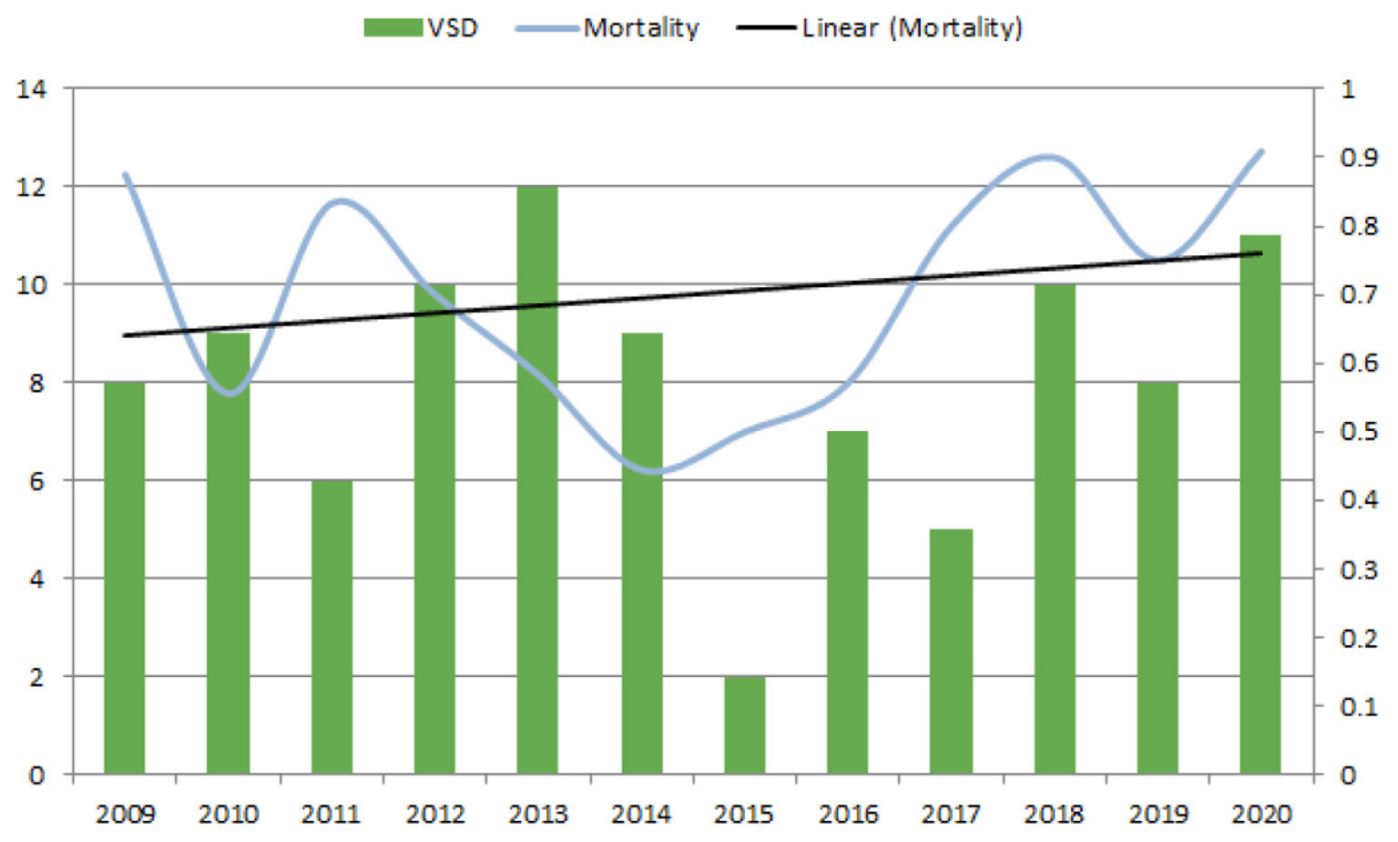
Annual VSD count between 2009 and 2020 and associated 1-year mortality. Left axis reports number of ischemic VSD per year. Right axis reports the ischemic VSD 1-year associated mortality. VSD, ventricular septal defect.

In-hospital and 1-year mortality rates were 71% and 86%, respectively (Figures 2, 3). A total of 28 patients (28.9%) were discharged from the hospital: their mean LVEF was 38% and in 7 cases (24%) a residual shunt was present.

Non-survivors at 1 year were older (74 vs. 63 yo, *p* < 0.001) with more severe organ dysfunction as reflected by higher lactate (2.8 vs. 1.4 mmol/l, *p* = 0.005) and natriuretic peptide (23 vs. 9 x N, *p* = 0.003) at admission. Non-survivors were more likely to have undergone inotrope/vasopressors support (67.6% vs. 28.6 %, *p* = 0.006) and less surgical repair (38.6% vs. 85.7%, *p* = 0.001) than survivors. The EuroSCORE II value was significantly higher for non-survivors (44.9% vs. 28% for survivors, *p* < 0.001). No significant difference was found according to medical history, VSD type, and position or type of aMCS (Supplementary Figure S1). The characteristics of patients according to in-hospital mortality are described in Supplementary Table S2.

Factors associated with higher in-hospital mortality in multivariate analysis were age [OR 1.06 (1.01–1.12) for each supplementary year, *p* = 0.02] and amines support requirement [OR 3.08 (1.22–11.4), *p* = 0.002], whereas surgery was associated with lower mortality [0.14 (0.04–045), *p* = 001] (Table 2).

Factors associated with 1-year mortality were age [1.06 (1.03–1.09), *p* < 0.001] and lactate [1.16 (1.09–1.23), *p* < 0.001], whereas surgery was associated with lower mortality [0.51 (0.28–0.94) *p* = 0.003] (Table 3). Additionally, we did not observe a decrease in 1-year mortality in the most recent period. On the contrary, we observed a trend of higher mortality (Figure 3).

**Table 3:** Factors associated with 1-year mortality by Cox regression analysis.

	**Univariate analysis**			**Multivariate analysis**		
	**HR**	**CI 95%**	***p* value**	**R**	**CI 95%**	***p* value**
Age (for each supplementary year)	1.05	[1.02–1.08]	< 0.01	0.06	[1.03–1.09]	< 0.01
Active smockers	0.46	[0.27–0.73]	< 0.01	-	-	-
Chronic kydney disease	0.48	[0.15–1.54]	0.22	-	-	-
**Biology**						
Lactates (for each supplementary mmol)	1.17	[1.11–1.24]	< 0.01	10.16	[1.09–1.23]	< 0.01
PTT (for each supplementary %)	0.98	[0.96–0.99]	< 0.01	-	-	-
Hepatic cytolysis (> 20 N)	1.52	[0.93–2.48]	0.09	-	-	-
**Management**						
VSD surgical repair	0.33	[0.20–0.55]	< 0.01	00.51	[0.28–0.94]	0.03
Amine use	1.99	[1.19–3.34]	< 0.01	-	-	-

CI, confidence interval; HR, hazard ratio; N, normal; PT, prothrombin time; VSD, ventricular septal defect.

For the 44 patients who underwent surgical septum repair, in-hospital and 1-year mortality were significantly lower than for medically managed patients (48% vs. 57% and 73% vs. 100%, respectively) (Figure 2). No significant difference was found among patients surgically managed on mean surgery delay or surgical technique used according to their in-hospital and 1-year vital status (Supplementary Table S3). No significant difference in survival was observed according to the use of aMCS or according to aMCS type (Supplementary Figures S1, S2).

## Discussion

Thanks to a 12-years retrospective analysis of our tertiary centre's experience, we demonstrated (1) the scarcity of post-ischaemic VSD (mean of 8 patients per year either <0.8% of ACS managed), (2) its severe prognosis close to 1 on 3 deceased at hospital discharge and up to 100% at 1 year in case of medical treatment, and (3) factors associated with worse prognosis were age, amine use, and lactate increase, (4) whereas surgery seems to be the only effective treatment with a 1-year survival of 27%.

As in a recent large retrospective multicentric registry (The CAUTION study *n* = 475 patients in 26 sites) (11), we found no prognosis improvement during the last decade despite refinements in interventional cardiology, heart surgery, mechanical circulatory support, and intensive care. Despite not reporting specific analysis by time period, we did not find any mortality difference between periods with the persistence of severe prognosis with in-hospital mortality around 80% and up to 100% 1-year mortality for medically managed patients.

This observation is surprising and there are questions regarding its causes. One possible explanation is the lack of expertise even in tertiary centers due to the scarcity of the disease (8 per year even in our reference center), preventing substantial practice enhancement. Another explanation is that today, the majority of patients arrive at our tertiary center contrary to before when they were probably less referred and died in primary or secondary centers. But, the absence of difference in terms of severity (data not shown) or incidence change during a 10-year period is not in favor of this. We only found a numerical VSD increase in 2020 which could be linked to the SARS-CoV-2 pandemic as previously explained, particularly by latency in management (12).

Another potential explanation is based on non-considered cofactors such as the systemic inflammatory response syndrome which could play an important role in the worsening of the associated MOF but also in the widening of the VSD. So, it might be a potential therapeutic target in these severe patients. But despite attractive pathophysiological hypotheses, inflammatory blocking or modulation (by anti-IL6, anti-TNF for example) and blood purification techniques (such as Cytosorb®) have yielded conflicting results (13, 14). They should only be discussed in the most severe patients as a rescue strategy based, for example, on a high level of plasma IL-6 or on the need for high doses of vasopressors, as recently suggested.

We reported a predominance of apical VSD with moderate RV dysfunction as previously reported (11). Interestingly, the VSD site was not prognostic in our study although the posterior position was associated with higher in-hospital mortality in the CAUTION study (11). The smaller population reported here may explain this difference. Due to the retrospective design of the study, TTE data were incomplete without standardization to evaluate and describe the RV function preventing any specific analysis.

As in previous reports (11), nearly half of patients were in cardiogenic shock at initial management (45%). The initial haemodynamic instability was related not only to the early prognosis but also to the patient's long-term prognosis, as highlighted by the multivariate analysis. This also confirms the prognostic weight of age at treatment. Only surgery was associated with a better prognosis (52% survival at hospital discharge and 27% at 1 year). This association was significant with in-hospital mortality [OR 0.14 (0.04–0.45), *p* < 0.001] and persisted with 1-year mortality [OR 0.51(0.28–0.94), *p* = 0.03] reaffirming the pivotal place of surgery in the management of post-MI VSD patients.

The place, type, and timing of aMCS could not be discussed with our cohort since the timing of insertion was not collected and the majority of patients received IABP (*n* = 68, 74%). Other devices were marginal (3 Impella® and 13 ECMO), preventing any conclusion contrary to the previous meta-analyses (15, 16). Dedicated studies may help the decision on timing and type of aMCS but these seem impossible to conduct due to the scarcity of this complication and the absence of consensual management even in a unique center. Direct LV and/or RV venting sounds attractive based on physiological and *ex-vivo* simulating studies but no specific studies to date report its use in these complex cases (17). Direct LV unloading is associated with a decrease in LVEDP and PCWP and an increase in coronary perfusion, suggesting higher potential LV recovery and preventing the worsening of the RV function associated with renal and hepatic failure (17). Nevertheless, the enthusiasm for direct ventricle venting could be moderated by the risk of worsening mechanical complications and systemic embolism due to its intra-cavity position. Further, all aMCS available are associated with potential adverse events in particular haemolysis, bleedings, limb ischaemia, and vascular complications (from 3.0% for IABP to 5.6% for Impella and 15.8% for ECMO) that potentially worsen the patient prognosis with a significant association with in-hospital mortality in a recent comparative observational study (56.3% with ECMO vs. 33.8% with Impella and 26.2% with IABP) (18). Despite frequent adverse events, the use of aMCS is sometimes mandatory in order to prevent the aggravation of cardiac and multi-organ failure and the death of the patient. The type and timing of device implantation are subject to debate without consensus to date. ECMO is probably the higher circulatory support and the best way to perfuse and oxygenate peripheral organs but several preclinical and clinical studies have shown the association between an increase in LV afterload and an increase in LVEDP and PCWP (19). LV overload seemed to correlate with the intensity of flow and was associated with the worst prognosis including mortality, especially in the case of LV failure (20, 21). In the case of VSD, these effects potentially lead to a worsening of the left-to-right shunt with an increased risk of RV dysfunction and death. Another possibility is to associate an early and systematic left heart decompression with an IABP association, a percutaneous balloon atrioseptostomy or a direct LV-unloading (22, 23). So, a combination of multiple aMCSs could improve peripheral organ perfusion while unloading the LV and RV. The combination of ECMO and Impella (ECMELLA), seems to provide the greatest degree of overall circulatory support while simultaneously unloading the LV (24) and may be a good alternative. But a combination of multiple aMCSs increases associated adverse events as recently demonstrated (24). This should be a case-by-case discussion in the absence of clear recommendations. In all cases, the choice should also depend on the aMCS availability and the multidisciplinary expertise and practice of the center. An important point seems to emerge from the literature, namely the early implantation of aMCS, whatever its type, aimed at preventing multiorgan organ failure and preserving RV and LV functions (11, 25).

Finally, the place of percutaneous closure is still debated, fuelled by contradictory literature. Patient selection and an expert team showed interesting results but, in our study, it was only proposed as a last line of treatment in very severe cases with a 100% in-hospital mortality. The low level of local expertise in this highly technical area may also have contributed to our poor results. Recently a large UK national registry (372 patients with post-MI VSD), showed that percutaneous closure has been increasingly used in clinical practice over recent years accounting for 31.6% of ischaemic VSD patients′ management alone and for 7.4% in association with surgical repair. They did not find a difference in long-term mortality between patients managed by surgical or percutaneous closure (61.1% vs. 53.7%, *p* = 0.17), but in-hospital mortality was lower in the surgical group (55.0% vs. 44.2%, *p* = 0.048). Interestingly, the percutaneous approach [aHR 1.44 (1.01–2.05), *p* = 0.04], and the number of vessels with coronary artery disease [aHR 1.22 (1.01–1.47), *p* = 0.043] were two of the three independent factors associated with long-term mortality, suggesting better results for the surgical approach (26). Surgery and percutaneous closure should not be opposed but may be combined since 16.1% of percutaneous patients subsequently had surgery and 7.8% of surgical patients subsequently had percutaneous treatment. This justifies a multidisciplinary approach in expert centers, allowing a patient-centered approach in terms of medical management, type of device, and timing of implantation, but also of VSD closure strategy.

### Limits

The retrospective and monocentric design of our study with a collection over 12 years explain most of the limits of our manuscript and preclude the conclusion or generalization of our results. The timing of aMCS implantation and explantation was not reported, preventing any analysis of its effect on outcomes. Moreover, the number of patients supported by aMCSs other than IABP [*n* = 15 with Impella (whatever its type) and/or ECMO] do not allow specific and independent analysis. Finally, no dedicated protocol was available to harmonize VSD patients′ management during the study period, explaining different types and timing of aMCS implantation, but also differences in medical management and surgical closure indication. Nevertheless, the persistence of high mortality of the post-ischemic VSD remains a real conclusion. The outstanding questions concern, on the one hand, the medical treatment put in place and the need to optimize and formalize it; and on the other hand, the question of patients who are contraindicated to surgery, which in fact condemns them to almost certain death (“self-fulfilling prophecy”). This reinforces the absolute necessity of implementing standardized multidisciplinary management and decision-making protocols.

## Conclusion

A post-ischaemic ventricular septal defect is an uncommon but still severe complication, involving <0.8% of all ACS and associated with an in-hospital mortality of 73% and 1-year mortality of 86%. Surgery remains the only treatment associated with better survival but is not feasible for all patients. Patient selection and the timing of surgery, as well as the type of aMCS in the waiting period or alternative therapies, should be addressed by larger multicentre dedicated studies.

## Data availability statement

The raw data supporting the conclusions of this article will be made available by the authors, without undue reservation.

## Ethics statement

Ethical review and approval was not required for the study on human participants in accordance with the local legislation and institutional requirements. Written informed consent for participation was not required for this study in accordance with the national legislation and the institutional requirements.

## Author contributions

Conceptualization: CD, HT, and FB. Data curation: HT, JP, CD, CB, FL, BM, OL, ME, MG, and CD. Formal analysis: FB, HT, and CD. Methodology and visualization: CD and FB. Project administration: CD and MG. Supervision: CD. Validation: HT, CD, FB, ME, and CD. Writing—original draft: HT and CD. Writing—review and editing: All authors. All authors contributed to the article and approved the submitted version.

## Conflict of interest

The authors declare that the research was conducted in the absence of any commercial or financial relationships that could be construed as a potential conflict of interest.

## Publisher's note

All claims expressed in this article are solely those of the authors and do not necessarily represent those of their affiliated organizations, or those of the publisher, the editors and the reviewers. Any product that may be evaluated in this article, or claim that may be made by its manufacturer, is not guaranteed or endorsed by the publisher.
