# Supplementary material for: Incidence, management, and prognosis of post-ischaemic ventricular septal defect: Insights from a 12-year tertiary centre experience
Source: Front Cardiovasc Med. 2022 Dec 6;9:1066308. doi: 10.3389/fcvm.2022.1066308 (PMC9763320; doi:10.3389/fcvm.2022.1066308)
Supplement: Supplementary file 1 [file Data_Sheet_1.DOCX]

**Incidence, management and prognosis of post-ischemic ventricular septal defect: insights from a twelve-year tertiary centre experience**.

Treille de Grand Saigne.H et al 2022

1. Supplementary Table 1: Description of the population of patients presenting an ischemic ventricular septal defect according to the use of an acute mechanical circulatory support.
2. Supplementary Table 2: Description of the population of patients presenting an ischemic ventricular septal defect according to their in-hospital mortality
3. Supplementary Table 3: Analysis of technics and delay for surgical VSD repair for patients with surgical VSD management (n = 44)
4. Supplementary Figure 1: Kaplan-Meier survival curve according to the use of an acute mechanical circulatory support (Red curve) or not (blue curve).
5. Supplementary Figure 2: Kaplan-Meier survival curve according to the type of acute mechanical circulatory support used: no mechanical circulatory support (blue line), IABP (green line) and others acute mechanical circulatory support (green line)

**Supplementary Table 1:** **Description of the population of patients presenting an ischemic ventricular septal defect according to the use of an acute mechanical circulatory support**

|  |  | | **All**  **(n=97)** | **aMCS**  **(n=70)** | **No aMCS**  **(n=27)** | **p-value** |
| --- | --- | --- | --- | --- | --- | --- |
|  | Age, years old | | 73 +/- 11 | 71 +/- 10 | 77 +/- 9 | 0.01 |
|  | Male sex. n (%) | | 54 (55.7) | 40 (57.1) | 40 (57.1) | 0.63 |
| Cardiovascular risk factors. n (%) | | | | |  |  |
|  | Smokers (n=95) | | 37 (38.9) | 27 (39.1) | 10 (38.5) | 0.95 |
|  | Diabetes (n=96) | | 30 (31.3) | 22 (31.4) | 8 (30.8) | 0.95 |
|  | Dyslipidemia (n=95) | | 32 (33.7) | 22 (31.9) | 10 (38.5) | 0.54 |
|  | Hypertension (n=95) | | 53 (55.8) | 35 (50.7) | 18 (69.2) | 0.1 |
|  | Obesity (BMI > 30) (n=90) | | 17 (18.9) | 14 (0.9) | 3 (13) | 0.4 |
| Medical history. n (%) | | |  |  |  |  |
|  | Ischemic cardiomyopathy (n=94) | | 9 (9.6) | 3 (11.5) | 6 (8.8) | 0.7 |
|  | PAD (n=95) | | 7 (7.4) | 2 (2.9) | 5 (20) | 0.02 |
|  | CKD (n=95) | | 6 (6.3) | 4 (5.8) | 2 (7.7) | 0.52 |
| Clinical characteristics at admission. n (%) | | | | | | |
|  | Right HF signs (n=87) | | 35 (40.2) | 27 (44.3) | 8 (30.8) | 0.24 |
|  | Left HF signs (n=92) | | 54 (58.7) | 38 (58.5) | 16 (59.3) | 0.94 |
|  | Hemodynamics instability (n=97) | | 53 (54.6) | 41 (58.6) | 12 (44.4) | 0.21 |
| Electrocardiogram at admission. n (%) | | | | | |  |
|  | STEMI (n=95) | | 75 (79) | 54 (79.4) | 21 (77.8) | 0.86 |
|  | Q wave | | 70 (73.7) | 50 (73.5) | 20 (74.1) | 0.95 |
| Angiocoronarography. n (%) | | |  |  |  |  |
|  | Ad-hoc revascularization | | 49 (50.5) | 33 (47.1) | 16 (59.3) | 0.28 |
|  | Significant other coronary artery disease (n=91) | | | | | 0.29 |
|  |  | One-vessel disease | 37 (40.7) | 31 (44.1) | 6 (27.3) |  |
|  |  | Two-vessel disease | 33 (36.3) | 24 ( 34.8) | 9 (40.9) |  |
|  |  | Tri-vessel disease | 21 (23.1) | 14 (20.3) | 7 (31.8) |  |
| Biology at admission | | |  |  |  |  |
|  | Lactates (mmol/l) (n=84) | | 2.65 [1.75 - 4.7] | 2.4 [1.4 - 4.6] | 3 [2 - 8.2] | 0.2 |
|  | PTT (%) (n=91) | | 66 +/- 19 | 63 +/- 19 | 75 +/- 19 | 0.01 |
|  | Hepatic cytolysis (n x Normale) (n=90) | | 6 [2 - 17] | 6 [2 - 20] | 5 [2 - 17] | 0.52 |
|  | Total bilirubin (mmol/l) (n=88) | | 21.8 +/- 13.6 | 23.3 +/- 14.3 | 17.5 +/- 10.6 | 0.07 |
|  | pH (n=91) | | 7.39 +/- 0.12 | 7.39 +/- 0.13 | 7.37 +/- 0.12 | 0.47 |
|  | Troponin (n x Normale) (n=95) | | 420 [217 - 1192] | 427 [180 - 1440] | 396 [238 - 900] | 0.82 |
|  | Natriuretic peptid (n x Normale) (n=66) | | 20 [9 - 49] | 20 [9 - 39] | 26 [7 - 58] | 0.91 |
|  | eGFR (MDRD) ml/min/1.73m² (n=96) | | 43 [28 - 73] | 43 [28 - 72] | 41 [30 - 75] | 0.77 |
| Echocardiography (TTE or TOE). n (%) | | | | | | |
|  | LVEF (%) (n=96) | | 44 +/- 13 | 44 +/- 14 | 43 +/- 12 | 0.6 |
|  | RV dilatation (n=88) | | 50 (56.8) | 41 (62.1) | 9 (40.9) | 0.08 |
|  | RV dysfunction (n=92) | | 44 (47.8) | 34 (50) | 10 (41.7) | 0.48 |
| VSD characteristics | | |  |  |  |  |
|  | Size by TTE (mm) (n=67) | | 14 +/- 8 | 15 +/- 7 | 10 +/- 4 | 0.06 |
|  | Surgical size (mm) (n=38) | | 23 +/- 13 | 26 +/- 13 | 15 +/- 5 | 0.03 |
| EUROSCORE 2 (n=95) | | | 42.4 +/- 20.7 | 40 +/- 19 | 48 +/- 23 | 0.13 |
| VSD management | | |  |  |  |  |
|  |  | Inotrops or vasopressors | 60 (61.9) | 50 (71.4) | 10 (37.4) | 0.002 |
|  |  | Surgical closure | 44 (45.4) | 36 (51.4) | 8 (29.6) | 0.05 |
|  |  | Percutaneous closure | 4 (4.1) | 3 (4.3) | 1 (3.7) | 0.89 |
|  |  | Heart transplantation | 1 (1) | 1 (1.4) | 0 (0) | 0.53 |
| Length of stay (days) (n=96) | | | | | | |
|  |  | ICU/ICCU LOS | 7 [3 - 12] | 9 [4 - 13] | 3 [2 - 7] | 0.002 |
|  |  | Total LOS | 8 [3 - 16] | 9 [4 - 19] | 4 [2 - 12] | 0.018 |

aMCS, acute mechanical circulatory support; BMI, body mass index; CKD, chronic kidney disease: Cx, circonflex coronary artery: eGFR, estimated glomerular filtration rate; HF, heart failure; IABP, intra-aortic balloon pump; ICU, intensive care unit; ICCU, intensive cardiac care unit; LAD, left anterior descending artery; LOS, length of stay; LVEF, left ventricular ejection fraction; MCS, mechanical circulatory support; PAD, peripheral arterial disease; PTT, prothrombin time; RCA, right coronary artery; RV, right ventricle: TOE; transesophagal echocardiography; TTE, transthoracic echocardiography; VA-ECMO, veno-arterial extracorporeal membrane oxygenation; VSD, ventricular septal defect

**Supplementary Table 2:** **Description of the population of patients presenting an ischemic ventricular septal defect according to their in-hospital mortality.**

|  |  | | | Global population (n=97) | | Non-survivors (n=69) | Survivors (n=28) | | p-value | |  |
| --- | --- | --- | --- | --- | --- | --- | --- | --- | --- | --- | --- |
|  | Age | | | 73 +/- 11 | | 75 +/- 9 | 68 +/- 13 | | 0.001 | |  |
|  | Male sex. years old | | | 54 (55.7) | | 37 (53.6) | 17 (60.7) | | 0.52 | |  |
| Cardiovascular risk factors, n (%) | | | | | | | | | | |  |
|  | Smokers (n=95) | | | 37 (38.9) | | 22 (32.8) | 15 (53.6) | | 0.06 | |  |
|  | Diabetes (n=96) | | | 30 (31.3) | | 23 (33.8) | 7 (25) | | 0.40 | |  |
|  | Dyslipidemia (n=95) | | | 32 (33.7) | | 21 (31.3) | 11 (39.3) | | 0.45 | |  |
|  | Hypertension (n=95) | | | 53 (55.8) | | 39 (58.1) | 14 (50.0) | | 0.46 | |  |
|  | Obesity (BMI > 30) (n=90) | | | 17 (18.9) | | 11 (17.4) | 6 (21.4) | | 0.67 | |  |
| Medical history, n (%) | | | |  | |  |  | |  | |  |
|  | Ischemic cardiomyopathy (n=94) | | | 9 (9.6) | | 6 (9.1) | 3 (107) | | 0.80 | |  |
|  | PAD (n=95) | | | 7 (7.4) | | 6 (8.8) | 1 (3.7) | | 0.38 | |  |
|  | CKD (n=95) | | | 6 (6.3) | | 3 (4.5) | 3 (10.7) | | 0.25 | |  |
| Clinical characteristics at admission. n (%) | | | | | | |  | |  | |  |
|  | Right HF signs (n=87) | | | 35 (40.2) | | 23 (36.5) | 12 (50) | | 0.25 | |  |
|  | Left HF signs (n=92) | | | 54 (58.7) | | 40 (59.7) | 14 (56.0) | | 0.74 | |  |
|  | Hemodynamic instability (n=91) | | | 53 (54.6) | | 40 (58) | 13 (46.4) | | 0.30 | |  |
| Electrocardiogram at admission, n (%) | | | | | | | | |  | |  |
|  | STEMI (n=95) | | | 75 (79) | | 54 (80.6) | 21 (75.0) | | 0.54 | |  |
|  | Q wave | | | 70 (73.7) | | 51 (76.1) | 19 (67.9) | | 0.41 | |  |
| Angiocoronarography, n (%) | | | |  | |  |  | |  | |  |
|  | Culprit lesion (n=91) | | |  | |  |  | | 0.28 | |  |
|  |  | LAD | | 51 (56) | | 33 (51.6) | 18 (66.7) | |  | |  |
|  |  | RCA | | 37 (40.7) | | 28 (43.8) | 9 (33.3) | |  | |  |
|  |  | Cx | | 3 (3.3) | | 3 (4.7) | 0 (0) | |  | |  |
|  | Ad Hoc revascularization (n=97) | | | 49 (50.5) | | 37 (53.6) | 12 (42.9) | | 0.33 | |  |
|  | Significant other coronary artery disease (n=91) | | | | | |  | | 0.75 | |  |
|  |  | One-vessel disease | | 37 (40.7) | | 26 (40.7) | 11 (40.7) | |  | |  |
|  |  | Two-vessel disease | | 33 (36.3) | | 22 (34.4) | 11 (40.7) | |  | |  |
|  |  | Tri-vessel disease | | 21 (23.1) | | 16 (25.0) | 5 18.5) | |  | |  |
| Biology at admission | | | |  | |  |  | |  | |  |
|  | Lactates (mmol/l) (n=84) | | | 2.65 [1.75 - 4.7] | | 3 [2 - 5.4] | 1.9 [1.2 - 2.6] | | 0.003 | |  |
|  | PTT (%) (n=91) | | | 66 +/- 19 | | 64 +/- 21 | 71 +/- 15 | | 0.09 | |  |
|  | Hepatic cytolysis (n x Normale) (n=90) | | | 6 [2 - 17] | | 8 [2 - 24] | 4 [2 - 11] | | 0.03 | |  |
|  | Total bilirubin (mmol/l) (n=88) | | | 21.8 +/- 13.6 | | 21.5 +/- 13.1 | 22.3 +/- 14.7 | | 0.80 | |  |
| Echocardiography (TTE or TOE), n (%) | | | | | | |  | |  | |  |
|  | LVEF (%) (n=96) | | | 44 +/- 13 | | 45 +/- 15 | 42 +/- 14 | | 0.48 | |  |
|  | RV dilatation (n=88) | | | 50 (56.8) | | 34 (55.7) | 16 (59.3) | | 0.75 | |  |
|  | RV dysfunction (n=92) | | | 44 (47.8) | | 34 (52.3) | 10 (37) | | 0.18 | |  |
| VSD characteristics | | | | | | | | | | |  |
|  | Size by TTE (mm) (n=67) | | | 14 +/- 8 | | 13 +/- 7 | 15 +/- 10 | | 0.37 | |  |
|  | Surgical size (mm) (n=38) | | | 23 +/- 13 | | 25 +/- 15 | 22 +/- 10 | | 0.48 | |  |
|  | VSD localisation, n (%) (n=96) | | | | | |  | | 0.42 | |  |
|  |  | Basal | | 28 (29.2) | | 22 (32.4) | 6 (21.4) | |  | |  |
|  |  | Median | | 16 (16.7) | | 12 (17.6) | 4 (14.3) | |  | |  |
|  |  | Apicale | | 52 (54.3) | | 34 (50) | 18 (64.3) | |  | |  |
| Euroscore 2 (n=95) | | | 42.4 +/- 20.7 | | 48.1 +/- 18.3 | | | 28 +/- 19.7 | | <0.001 | |
| VSD management, n (%) | | | |  | |  |  | |  | |  |
|  |  | Inotrops or vasopressors | | 60 (61.9) | | 49 (71) | 11 (39.3) | | 0.004 | |  |
|  |  | IABP | | 60 (61.9) | | 41 (59.4) | 19 (67.9) | | 0.43 | |  |
|  |  | VA-ECMO | | 13 (13.4) | | 10 (14.5) | 3 (10.7) | | 0.62 | |  |
|  |  | Impella® | | 3 (3.1) | | 0 (0) | 3 (10.7) | | 0.006 | |  |
|  |  | All acute MCS | | 70 (72.2) | | 48 (69.6) | 22 (78.6) | | 0.37 | |  |
|  |  | Surgical closure | | 44 (45.4) | | 21 (30.4) | 23 (82.1) | | <0.001 | |  |
|  |  | Percutaneous closure | | 4 (4.1) | | 4 (5.8) | 0 (0) | | 0.19 | |  |
|  |  | Heart transplantation | | 1 (1) | | 0 (0) | 1 (3.6) | | 0.11 | |  |
|  | Length of stay (n=96) | | |  | |  |  | |  | |  |
|  |  | ICU/ICCU LOS | | 7 [3 - 12] | | 5 [2 - 9] | 13 [9 - 27] | | <0.001 | |  |
|  |  | Total LOS | | 8 [3 - 16] | | 5 [2 - 9] | 24 [15 - 35] | | <0.001 | |  |

BMI, body mass index; CKD, chronic kidney disease: Cx, circonflex coronary artery: eGFR, estimated glomerular filtration rate; HF, heart failure; IABP, intra-aortic balloon pump; ICU, intensive care unit; ICCU, intensive cardiac care unit; LAD, left anterior descending artery; LOS, length of stay; LVEF, left ventricular ejection fraction; MCS, mechanical circulatory support; PAD, peripheral arterial disease; PTT, prothrombin time; RCA, right coronary artery; RV, right ventricle: TOE; transesophagal echocardiography; TTE, transthoracic echocardiography; VA-ECMO, veno-arterial extracorporeal membrane oxygenation; VSD, ventricular septal defect

**Supplementary Table *3*: Analysis of technics and delay for surgical VSD repair for patients with surgical VSD management (n = 44)**

| Patients with surgical management of VSD | Global population (n=44) | Non survivors (n=21) | Survivors  (n=23) | p-value |
| --- | --- | --- | --- | --- |
| Time before surgical management (days) | 7 [4 - 10] | 7 [3 - 9] | 6.5 [5 - 12] | 0.27 |
| Atriotomy. n (%) | 2 (4.5) | 1 (4.8) | 1 (4.4) | 0.94 |
| Right ventriculotomy. n (%) | 35 (79.6) | 17 (81) | 18 (78.3) | 0.82 |
| Left ventriculotomy. n (%) | 42 (95.5) | 19 (90.5) | 23 (100) | 0.13 |

VSD. ventricular septal defect

**Supplementary Figure 1: Kaplan-Meier survival curve according to the use of an acute mechanical circulatory support (Red curve) or not (blue curve).**

On 97 patients. 11 patients were lost of follow-up and not included in survival analysis. Log-rank test p-value = 0.21**.** AMCS. acute mechanical circulatory support which include IABP. venoarterial ECMO and Impella® device.

**Supplementary Figure 2: Kaplan-Meier survival curve according to the type of acute mechanical circulatory support used: no mechanical circulatory support (blue line). IABP (red line) and others acute mechanical circulatory support (green line)**

On 97 patients. 11 patients were lost of follow-up and not included in survival analysis. Others mechanical circulatory support include venoarterial ECMO and Impella® device. Log-rank test p-value = 0.27.
